# Metabolic Engineering of the Phenylpropanoid and Its Primary, Precursor Pathway to Enhance the Flavor of Fruits and the Aroma of Flowers

**DOI:** 10.3390/bioengineering2040204

**Published:** 2015-11-27

**Authors:** Hadas Peled-Zehavi, Moran Oliva, Qingjun Xie, Vered Tzin, Michal Oren-Shamir, Asaph Aharoni, Gad Galili

**Affiliations:** 1 Department of Plant and Environmental Sciences, The Weizmann Institute of Science, Rehovot 76100, Israel; E-Mails: hadas.zehavi@weizmann.ac.il (H.P.-Z.); olivamor@weizmann.ac.il (M.O.); Qingjun.Xie@weizmann.ac.il (Q.X.); 2 Department of Ornamental Horticulture, Agriculture Research Organization, The Volcani Center, Beit Dagan 75359, Israel; E-Mail: vhshamir@volcani.agri.gov.il; 3 The Boyce Thompson Institute for Plant Research, Ithaca, NY 14853, USA; E-Mail: vt223@cornell.edu

**Keywords:** aroma and flavor biotechnology, metabolic engineering, aromatic amino acids, volatiles, secondary metabolism

## Abstract

Plants produce a diverse repertoire of specialized metabolites that have multiple roles throughout their life cycle. Some of these metabolites are essential components of the aroma and flavor of flowers and fruits. Unfortunately, attempts to increase the yield and prolong the shelf life of crops have generally been associated with reduced levels of volatile specialized metabolites and hence with decreased aroma and flavor. Thus, there is a need for the development of new varieties that will retain their desired traits while gaining enhanced scent and flavor. Metabolic engineering holds great promise as a tool for improving the profile of emitted volatiles of domesticated crops. This mini review discusses recent attempts to utilize metabolic engineering of the phenylpropanoid and its primary precursor pathway to enhance the aroma and flavor of flowers and fruits.

## 1. Introduction

**Figure 1:**
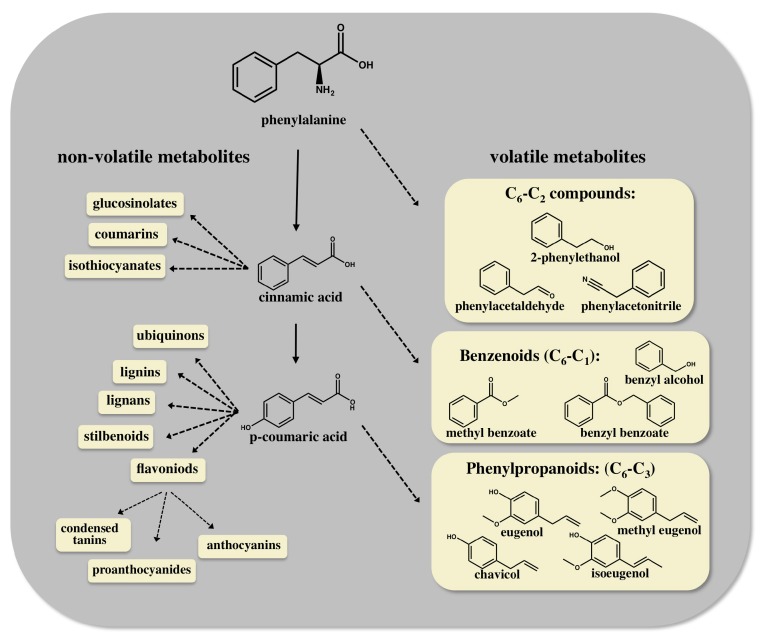
Schematic diagram of the phenylpropanoid/benzenoid biosynthetic pathway in plants. Phenylalanine, which is synthesized in the plastid through the shikimate and aromatic amino acids biosynthetic pathways, is the precursor for both volatile (right hand side) and non-volatile (left hand side) specialized metabolites. The chemical structure of representative volatile compounds that contribute to the aroma and flavor of flowers and fruits is shown. Dashed arrows represent several enzymatic steps.

Plants synthesize a diverse repertoire of specialized metabolites that play important roles in various aspects of plant life such as growth, development, reproduction, defense and environmental responses [1,2]. Volatile plant metabolites are lipophilic, low molecular weight compounds with high vapor pressure at ambient temperatures**,** which are known to be emitted to the environment by flowers, fruits, vegetative tissues and roots. These volatile metabolites can attract pollinators and seed dispersals, stimulate or suppress signaling cascades and protect against harsh environmental conditions, herbivores or pathogens [3,4,5]. They also contribute to the aroma and flavor of flowers and fruits [6,7,8]. The biosynthetic pathways of specialized metabolites evolve from primary metabolic routes, and they can be divided into several groups, including terpenes, fatty acid derivatives, carbohydrate derivatives, and amino acid derivatives. Aromatic amino acids (AAA)-derived specialized metabolites, which are the focus of this mini-review, are a major group of metabolites that originate from tyrosine, tryptophan and in particular from phenylalanine. They include many of the benzenoid and phenylpropanoid volatiles that confer enhanced aroma and flavor to flowers and fruits (Figure 1) [6,9,10].

Throughout the years, crop-breeding schemes were largely focused on attaining traits such as rapid growth, high yields and long post-harvesting shelf life. Less attention was devoted to maintaining or improving desirable aroma and flavor, partially because growers are generally paid for yield rather then for taste or aroma. Furthermore, aroma and flavor are complex traits controlled by multiple genes, and unlike yield or shelf life, are difficult to screen for. Hence, modern breeding schemes often lead to domesticated crops that produce less volatile metabolites, resulting in the loss of some of their desired aroma and flavor [7,9]. In recent years, metabolic engineering emerged as a practical tool to boost or change the production of volatile metabolites in the whole plant or in specific organs. Successful manipulation of the plant volatile metabolic profile requires an understanding of the relevant biosynthetic pathways and identification of the involved intermediates and products. Many of the genes and enzymes involved in the synthesis of AAA and especially of phenylalanine-derived volatile compounds are now characterized in model crop plants, as well as in several other plants bearing aromatic fruits and flowers [6,8]. Furthermore, several transcription factors involved in the regulation of flux through the phenylpropanoid/benzenoid pathway have been characterized [11,12]. However, much is still unknown about the regulation of the different biosynthetic branches of phenylpropanoids and the regulatory cross talk between them

## 2. Enhancing Aroma and Flavor by Metabolic Engineering of the AAA Derived Pathways

Several attempts have been made to improve the aroma and flavor of flowers and fruits by metabolic engineering of AAA-derived pathways (for recent extensive reviews see [6,8]). One possible strategy is the modification of an existing pathway or the introduction of a new branch in a pathway, for example by overexpressing an existing enzyme or a heterologous enzyme that directly leads to volatile synthesis. Alternatively, a competing pathway can be blocked, thereby channeling precursors to the desired pathway. Manipulation of transcriptional regulation can also be used to change the metabolic profile of AAA-derived volatiles. For example, the constitutive overexpression in tomato of members of the amino acid decarboxylases family, which perform the first and rate limiting step in the synthesis of phenylalanine-derived volatiles, resulted in the production of fruits with up to tenfold increased levels of 2-phenylacetaldehyde, 2-phenylethanol and 1-nitro-2-phenylethane [13]. In cultivated strawberry fruits, the carbon flux was redirected from anthocyanin pigment biosynthesis to the production of phenylpropene volatiles [14,15]. This was achieved through the downregulation of chalcone synthase, a key enzyme for the pathway branch leading to anthocyanin biosynthesis, simultaneously with the heterologous overexpression of a basil eugenol synthase or a petunia isoeugenol synthase gene. As a result, the levels of eugenol, isoeugenol, and related compounds were enhanced by orders of magnitude in comparison to their odor thresholds [14]. Introduction of *Arabidopsis* Production Of Anthocyanin Pigment1 (PAP1) transcription factor into rose flowers resulted in increased levels of phenylpropanoid-derived color and scent compounds, as well as higher levels of terpenoid scent compounds. Olfactory assays revealed that the enhanced levels of scent volatiles could be identified by both bees and humans [16]. However, the results of many of these metabolic engineering efforts demonstrated that as our knowledge of the biosynthetic pathways is far from being complete, the outcome of these modifications is often hard to predict. Enhancement of the desired volatiles could be hindered by the availability of the required substrates [17,18,19] or their modification into a non-volatile form [20]. Furthermore, the modification or addition of a single gene does not necessarily changes the overall flavor and aroma of the flower or the fruit, and it may be necessary to modify multiple targets to produce the desirable effect [21].

## 3. Enhancing Aroma and Flavor by Metabolic Engineering of AAA Biosynthetic Pathways

A different approach, taken by our lab in the last few years, is to augment the amount of the primary metabolites—the AAAs—in an effort to increase the carbon flux towards the metabolic synthesis of specialized metabolites. This approach can be used to study the regulatory interactions between pathways of primary and specialized metabolism associated with the AAAs, to identify bottlenecks in the biosynthetic pathways and to look at cross regulation between different pathway branches. Moreover, elucidation of the secondary metabolites produced by various plant species, particularly crop plants, is hindered by the fact that many of these metabolites accumulate to very low steady state levels that are below detection threshold. Enhancing the production, and hence the levels of AAA-derived specialized metabolites in transgenic lines, can serve as a valuable tool for the detection of these low abundance compounds.

### 3.1. Targeting the Shikimate Pathway (AroG*)

**Figure 2:**
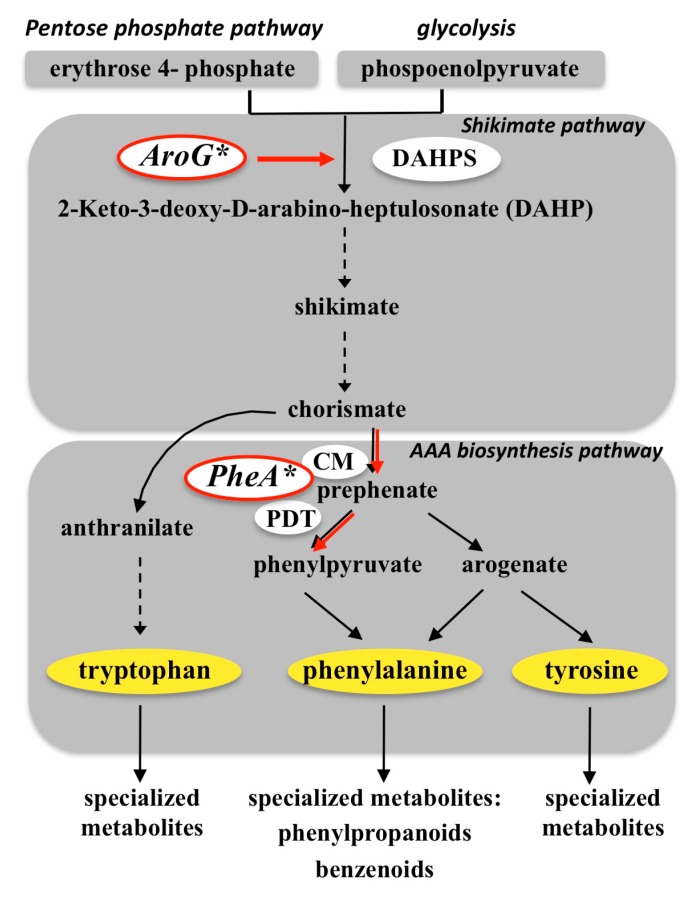
Schematic diagram of the shikimate and aromatic amino acid biosynthetic pathways in plants. Only some of the enzymes are depicted. Dashed black arrows represent several enzymatic steps. Red arrows represent enzymatic steps carried by the heterologously expressed bacterial enzymes AroG* and PheA*. DAHPS, 3-deoxy-d-arabino-2-heptulosonate 7-phosphate synthase; CM, chorismate mutase; PDT, prephenate dehydretase.

The synthesis of the three AAAs begins by utilizing the products of glycolysis and the pentose pathway, namely phosphoenolpyruvate and erythrose 4-phosphate. The first committed enzyme of the shikimate pathway, 3-deoxy-di-arabino-heptulosonate 7-phosphate synthase (DAHPS), condenses these two products to form 3-deoxy-di-arabino-heptulosonate 7-phosphate (DAHP). DAHP is then directed via the shikimate and the AAAs biosynthesis pathways to form AAA, with chorismate serving as an intermediate metabolite (Figure 2). Thus, the shikimate pathway serves as a metabolic bridge between primary and secondary metabolism with regard to the regulation of AAA biosynthesis [10]. In an attempt to study the effect of enhanced precursor levels on the accumulation of specialized metabolites in different plants, and in particular to study the effect on the accumulation of volatile compounds that contribute to aroma, we have employed a metabolic engineering approach. Bacterial feedback-insensitive variants of biosynthetic enzymes were utilized in order to bypass enzyme feedback inhibition loops of the shikimate and AAAs biosynthesis pathways, and to enhance the carbon flux through the pathway. This approach was first utilized on the model plant *Arabidopsis thaliana* [22,23], and then implemented in crop plants with petunia flowers used as a model for fragrant flowers [24] and a tomato variety that naturally possesses long shelf life, but has a relatively low flavor/aroma value used as a fruit model [25,26]. Recently, this approach was also implemented in a cell suspension derived from red grape berries [27]. One heterologous bacterial gene that was expressed in the transgenic lines is a mutant bacterial *AroG* gene (AroG*), encoding a feedback-insensitive DAHPS (Figure 2). As DAHPS is the first committed enzyme of the shikimate pathway, condensing phosphoenolpyruvate and erythrose 4-phosphate to form DAHP, it plays a major role in the conversion of primary carbon to all three AAAs and from them to specialized metabolites. Indeed, overexpression of the AroG* gene in the different plants generally led to increased accumulation of AAAs and to enhanced accumulation of the specialized metabolites derived from them. In petunia, the constitutive overexpression of AroG* under a strong promoter resulted in significantly higher levels of the aromatic amino acid phenylalanine in the flower petals of the transgenic plants, as well as enhanced levels of various fragrant benzenoid-phenylpropanoid volatile compounds that produce scent-associated molecules [24]. These changes in the levels of specialized metabolites caused a noticeable increase in the floral scent of the petunia flower while no change was observed in the longevity of the flowers. Interestingly, additional results suggested that phenylalanine produced in leaves could be transported through the stem to the flowers and serve as a precursor for additional formation of multiple fragrant metabolites that produce attractive scent [24]. Similar to the petunia plants, AroG* expression in tomato fruits under a fruit ripening-specific promoter, influenced the levels of primary as well as specialized metabolites [25]. The transgenic tomato fruits had increased levels of shikimic acid and AAAs, as well as multiple volatile and non-volatile phenylpropanoid specialized metabolites and carotenoids. Importantly, an organoleptic test, performed by trained panelists, suggested that the ripe transgenic tomato fruits had a preferred floral aroma compared with fruits of the wild-type line [25]. In a grape cell suspension, overexpressing the AroG* gene under constitutive promoter led to high accumulation of tyrosine and phenylalanine. Specialized metabolites that are naturally synthesized by these cells such as resveratrol, which is associated with the health-inducing properties of red grapes, were significantly enhanced [27]. Taken together, these results demonstrate that enhancing the carbon flux towards the AAA synthetic pathway will indeed increase the levels of AAAs and their precursors, as well as the levels of specialized metabolites, and can contribute to the enhancement of aroma. The exact profile of enhancement is dependent on the existing/active metabolic pathways in the plant and the tissue in which the genes are expressed. Thus, comparing the outcome in different plants and tissues can provide valuable metabolic information.

### 3.2. Targeting Phenylalanine Biosynthesis (PheA*)

A second bacterial gene heterologously expressed in transgenic plants is a mutated *PheA* gene (PheA*), encoding a feedback-insensitive bi-functional chorismate mutase/prephenate dehydratase gene. The *PheA* gene converts chorismate via prephentate into phenylpyruvate, directing the carbon flux to the phenylpyruvate branch of the AAAs pathway leading to phenylalanine synthesis (Figure 2). Thus, expression of PheA* was expected to direct the flux more specifically towards phenylalanine and the specialized metabolites that are derived from it [10]. Expression of the PheA* gene in Arabidopsis seedlings resulted in increased levels of phenylalanine [22]. Intriguingly, it affected the levels of specialized metabolites derived from all three AAAs. The PheA* overexpressing lines had increased levels of several specialized metabolites derived from phenylalanine and tyrosine, but reduced levels of several specialized metabolites derived from tryptophan. Though the mechanistic reason for these changes is still unclear, these results imply regulatory cross-interactions between the flux of AAA biosynthesis from chorismate and their metabolism into various specialized metabolites [22]. In contrast, expressing PheA* in tomato fruits led only to minor changes in the levels of the AAAs or any of the identified volatile metabolites [26], and tomato fruits of transgenic plants expressing both the AroG* and the PheA* genes had a metabolic profile that was predominantly impacted by the expression of the AroG* gene. However, the aroma attributes of the tomato fruits expressing both genes were unique and different from those of the lines expressing a single gene, suggesting a contribution of the PheA* gene to the overall metabolic profile [26]. Taken together, these recent studies suggest that enhancing the flux from primary metabolism towards specialized secondary metabolites is a promising metabolic engineering approach that enables the identification and possible removal of bottlenecks in the biosynthesis of specialized metabolites, and the enhancement of aroma in both flowers and fruits. This conclusion is supported by a recent study looking at the outcome of expressing the *Arabidopsis* transcription factor AtMYB12 in tomato fruits [28]. AtMYB12 is a transcription factor regulating flavonol biosynthesis in *Arabidopsis.* Expression of AtMYB12 in tomato fruits induced the expression of genes encoding enzymes of flavonol and hydroxycinnamic ester biosynthesis, resulting in exceptionally high levels of phenylpropanoids, specifically flavonols and caffeoyl qunic acids [29]. Interestingly AtMYB12 also induced the expression of genes encoding enzymes of primary metabolism, including DAHPS [28]. It was suggested that the increase in phenylpropanoids in AtMYB12 expressing tomatoes is the combined result of the transcriptional activation of genes encoding enzymes of secondary metabolism and the reprograming of carbon flux towards AAA biosynthesis through changes in primary metabolism. Thus, further improvement of aroma and flavor might be achieved by combining the enhanced flux from the shikimate and AAAs biosynthetic pathways with additional manipulations in downstream enzymes or branch pathways of specialized metabolites to further direct the flux towards specific desirable volatile metabolites.

## 4. Conclusions

In conclusion, the use of metabolic engineering can provide new information on the regulation of the channeling of primary metabolism to specialized metabolism, the cross-interaction of different metabolic pathways and the repertoire of specialized secondary metabolites in different plant species. In turn, gaining information on the various secondary metabolites and the metabolic pathways involving their production can contribute to the development of knowledge-based breeding strategies. Furthermore, metabolic engineering opens a new venue to stimulate the production of specialized metabolites and to enhance the quality of various plant organs. In particular, it can overcome the general negative correlation between flavor and aroma and long shelf life in different crops, an issue that has so far been difficult to solve by classical breeding.
